# Ameloblastomatous CCOT: A Case Report of a Rare Variant of CCOT with a Review of the Literature on Its Diverse Histopathologic Presentation

**DOI:** 10.1155/2013/407656

**Published:** 2013-10-09

**Authors:** Shailesh Menat, Shylaja MD, Kailash Attur, Kaushal Goyal

**Affiliations:** ^1^Department of Oral and Maxillofacial Surgery, Narsinhbhai Patel Dental College and Hospital, Visnagar, Gujarat 384315, India; ^2^Department of Oral Pathology and Microbiology, Narsinhbhai Patel Dental College and Hospital, Visnagar, Gujarat 384315, India; ^3^Department of Conservative and Endodontics, Narsinhbhai Patel Dental College and Hospital, Visnagar, Gujarat 384315, India

## Abstract

Calcifying odontogenic cyst is considered as a rare lesion and accounts for 1% of jaw cysts. It represents a heterogeneous group of lesions which exhibit a variety of clinicopathologic and behavioral features. It has been categorized as cyst and neoplasm. Even after several classification and subclassification, COC remains an enigma. WHO classification 2005 has reclassified the lesion as calcifying cystic odontogenic tumor (CCOT). Ameloblastomatous COC is a rare variant which is not much described in the literature. This report describes one such case which was large multicystic, involved the coronoid and condylar process of the mandible, and treated by subhemimandibulectomy. The case was recurrence free even after 1 year of followup.

## 1. Introduction

Calcifying odontogenic cyst (COC) was first described by Gorlin et al. (1962, 1964); hence, the eponym of “Gorlin cyst” is frequently used. The lesion has been widely occurring both peripherally and centrally in the jaws [1]. Although it was recognized as a distinct pathologic entity at first, COC shows extreme diversity in its clinical and histopathological features as well as its biological behavior [2]. 

COC was considered as a developmental odontogenic cyst with diverse origin [3]. It is a rare odontogenic pathology and constitutes to about from 0.37% to 2.1% of all odontogenic tumors [4] and about 1% of the jaw cysts reported [5]. A majority of COC are cystic in architecture and appear to be nonneoplastic, but they sometimes appear as a solid lesion; at least some of the solid lesions are neoplastic in nature [2].

According to the new WHO classification in 2005, COC has now been reclassified as calcifying cystic odontogenic tumor (CCOT) [6]. It often occurs in association with other odontogenic tumors such as ameloblastoma and complex odontoma [7]. The classification advocated by Hong et al. has two categories for CCOT associated with ameloblastoma: the ameloblastomatous cystic and the neoplastic variants associated with ameloblastoma.

Herewith, we are reporting a case of ameloblastomatous CCOT which will add one more rare case to the literature which might help in understanding the biologic behavior of this type of lesion.

## 2. Case Report 

A 20-year-old male patient visited the department of oral and maxillofacial surgery with a chief complaint of swelling on lower left 1/3 of face and disfigurement of face for 2 years.

Swelling which started gradually increased to attain the present size. 

**Figure 1:**
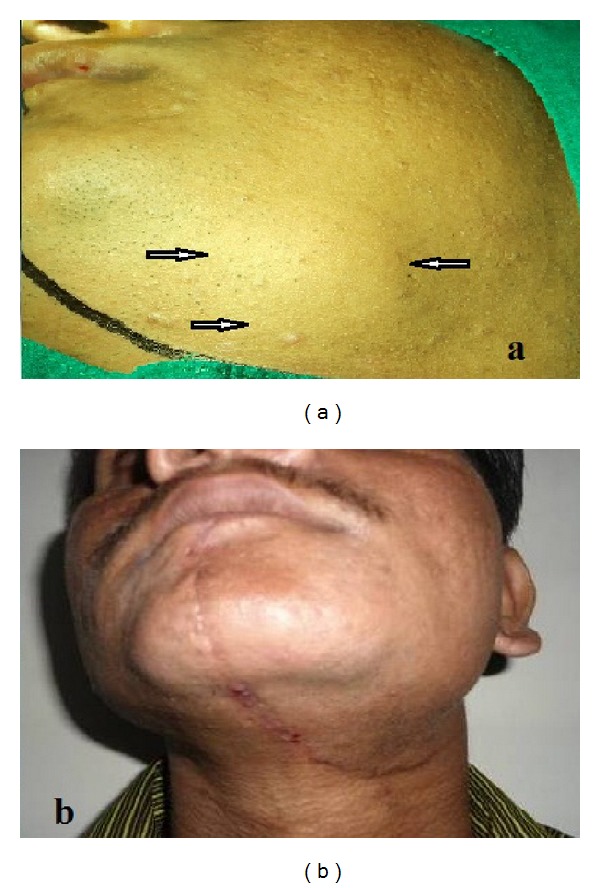
(a) Extraoral picture of the patient showing swelling in the left submandibular region. (b) Extraoral picture after 1 year of followup.

Extraoral examination revealed a swelling in the lower left back tooth region of the jaw and on the angle of the mandible (Figure 1(a)). Anteroposteriorly, the swelling extended from 2 cms distal to the angle of mouth to the ramus of mandible. Supero-inferiorly, it extends from 3 cms below the zygomatic arch to 1 cm beyond the lower border of the mandible.

Intraoral examination revealed a large swelling extending from mandibular left first molar to the ramus of the mandible, obliterating buccal vestibule and causing bucco-lingual expansion of the bone. Mucosa overlying the lesion was intact. 

**Figure 2:**
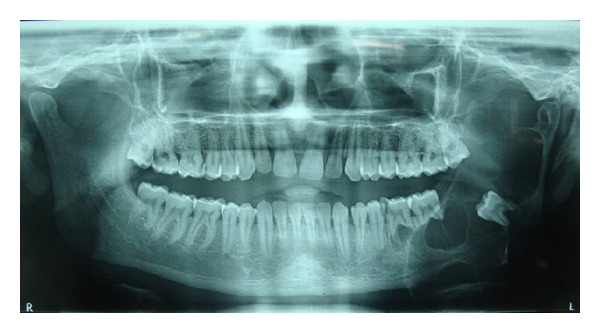
OPG showing multilocular radiolucency in relation to left third molar involving the angle, ramus, and coronoid process of the mandible. Root resorption is evident in mandibular I, II, and III molar.

The orthopantomograph revealed a multilocular radiolucency on the left side of the mandible extending from molar upto the condyle and coronoid area (Figure 2). The lesion contained the unerupted third molar dislocated inferiorly at the angle of mandible. Root resorption of second molar was evident.

**Figure 3:**
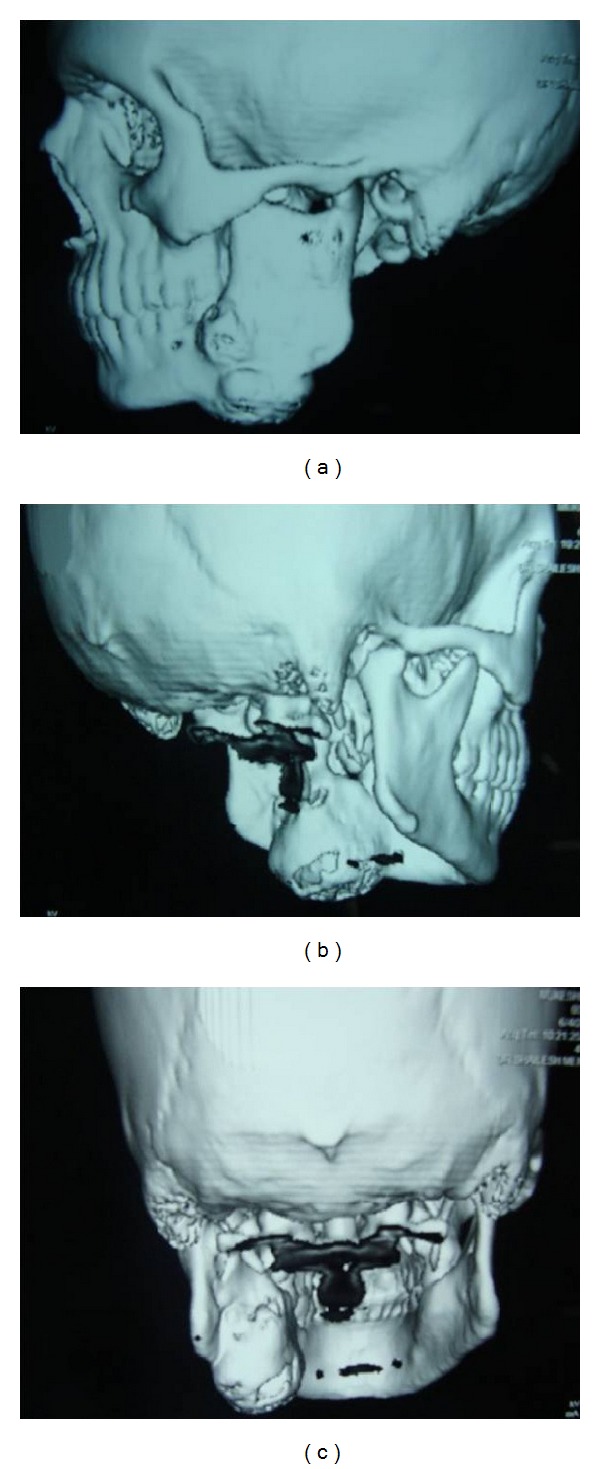
3D CT image showing the lesion in the body and ramus of the mandible, (a) left lateral surface, (b) lingual surface of left mandible, and (c) lingual surface of mandible from the posterior view.

CT scan showed a multiloculated, large cystic, and expansile lesion involving left ramus of mandible with significant cortical thinning. The solid tumor measured about 2.6 × 3.6 cm in axial plane with craniocaudal extension measuring 7.3 cm (Figure 3).

Based on history clinical feature and radiographic appearance, a provisional diagnosis of ameloblastoma or odontogenic keratocyst was made. An incisional biopsy was obtained from the left retromolar area to establish final diagnosis. Histopathologic examination revealed follicles with peripheral palisading cells with reverse polarity of nucleus and central stellate reticulum-like cells. Cystic degeneration within the follicles was also evident, suggestive of cystic ameloblastoma.

**Figure 4:**
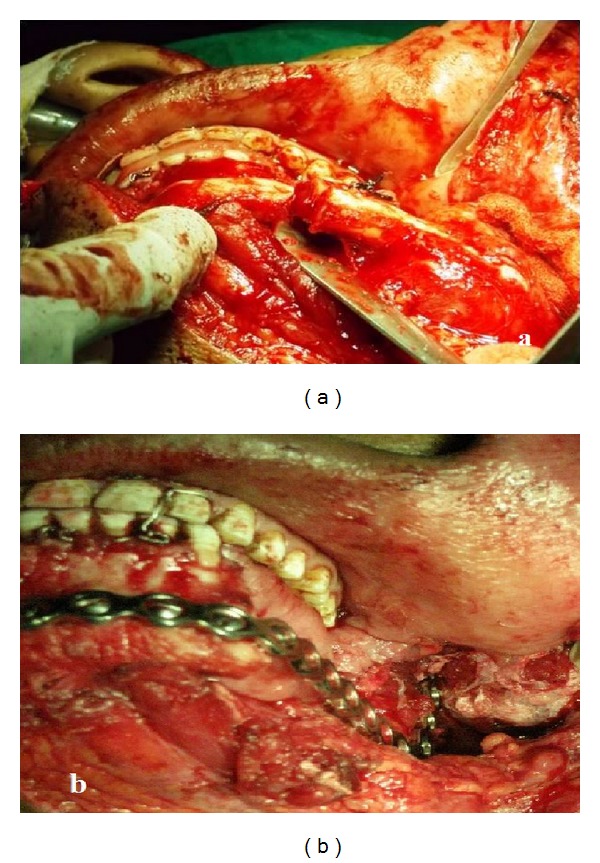
(a) Surgical photograph showing hemimandibulectomy and (b) condylar reconstruction plate.

 The patient was taken for surgery under general anaesthesia, and the lesion was reached through lip split submandibular incision. Hemimandibulectomy with disarticulation was performed, and condylar reconstructive plate was fixed in the area (Figures 4(a) and 4(b)). The resected specimen was sent for histopathological examination.

**Figure 5:**
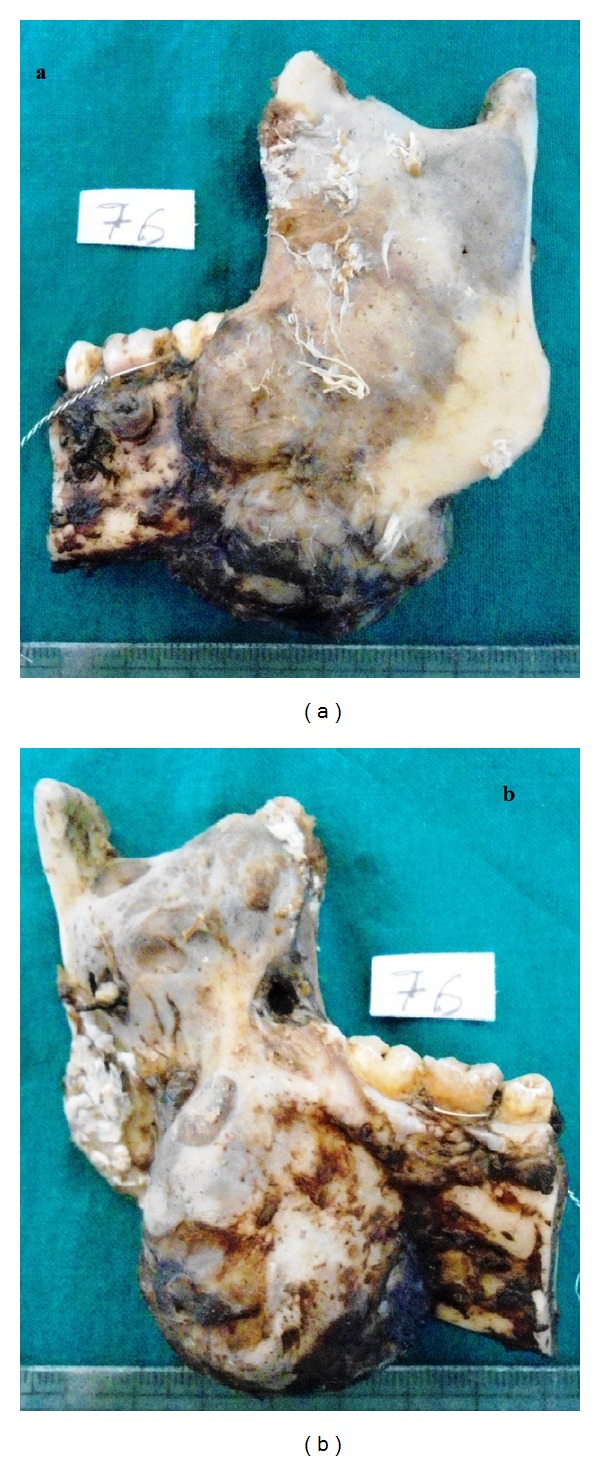
Resected mandible sent for histopathologic examination, (a) lateral surface, and (b) medial surface of the mandible.

The hemimandibulectomy specimen showed the resection margin till the mandibular left second premolar (Figures 5(a) and 5(b)). The bony margin was cut and taken for decalcification to know the adequacy of the surgery. Tissues were taken from multiple areas. The specimen was grossly cystic, but solid areas were seen on the lingual portion of the resected specimen. Bony tissues were taken for decalcification with 5% nitric acid. Soft tissues and decalcified tissue were routinely processed, and 4 *μ* thick sections were cut and stained with Hematoxylin and Eosin.

**Figure 6:**
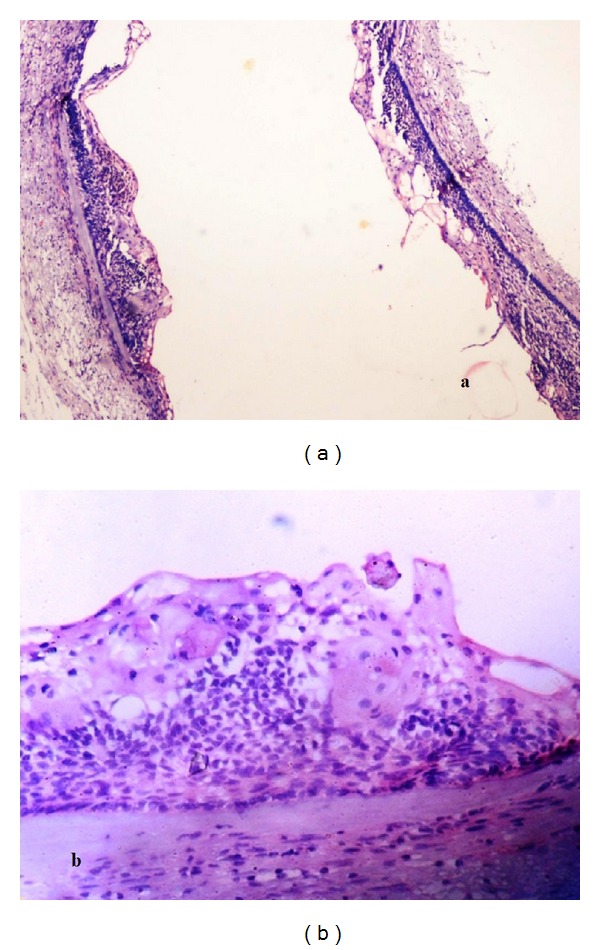
H & E stained section showing cystic lining with ghost cells and stroma with dysplastic dentin, (a) magnification ×40, (b) magnification ×400.

The section showed a cystic lining overlying fibrous stroma. The lining epithelium had flat to cuboidal basal cells with stellate superficial cells. Few areas showed tall columnar basal cells with reverse polarity of nucleus. In few areas, the stellate cells showed squamous differentiation. Few large eosinophilic, anucleated cells were seen with indistinct cell membrane suggestive of ghost cells (Figure 6). Few ghost cells showed features of calcification. Epithelium budding into the connective tissue was evident in many areas.

**Figure 7:**
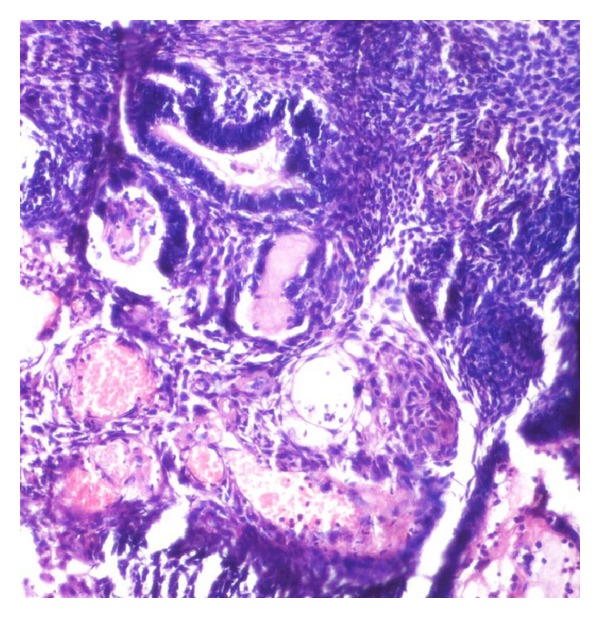
Odontogenic island in the stroma showing ghost cells.

Stroma showed thick bundles of collagen fibers with spindle fibroblasts. Juxta-epithelial hyalinization was seen suggestive of dysplastic dentin (Figures 6(a) and 6(b)). Proliferative odontogenic islands with ghost cells were also seen in the connective tissue capsule (Figure 7). Daughter cysts with ghost cells were also evident. Few areas showed dysplastic dentin in and around some odotogenic islands.

The resected bony margin was clear and free of any infiltration.

With the above histopathologic features, a diagnosis of ameloblastomatous CCOT was made. 

**Figure 8:**
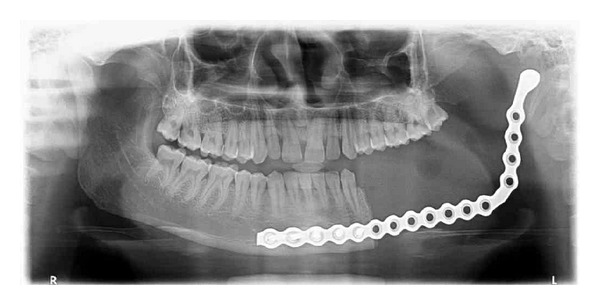
Posttreatment OPG after 1 year.

The patient has been under regular followup and has not exhibited any signs of recurrence after 1 yr of followup (Figure 1(b), Figure 8).

## 3. Discussion

CCOT was first described in 1932 by Rywkind who reported a lesion of the jaw which resembled cholesteatoma of the ear and thereafter called it as cholesteatoma of the jaw. In 1946, Thoma and Goldman described a lesion which they called a strange variant of ameloblastoma [7]. It was in 1962 that Gorlin first described it. 

In recent classification of the World Health Organization (2005), the term calcifying cystic odontogenic tumor (CCOT) has been replaced with calcifying odontogenic cyst (COC) that constitutes a benign cystic neoplasia that presents an epithelium with ghost cells which may display calcification in it [8].

CCOT is common in the second decade of life. Buchner and Pretorius have drawn attention to a bimodal age distribution with a second peak in the 6-7th decade of life. The youngest recorded patient was a 1 year old, the oldest an 82 years old [1]. There is a distinct peak in the second decade [9]. There are no particular predilections for either the maxilla or mandible, although the cases in the maxilla are more often in older patients. This lesion tends to occur in the canine-incisor portion in both jaws, but those in the maxilla occur more often at the anterior portion than those in the mandible [10]. Freedman et al. (1975) pointed out that 70% of their sample occurring in patients before the age of 41 were in the maxilla, whereas 80% in patients older than 41 were in the mandible [1]. 

According to Shamaskin et al., central COCs occur more commonly than peripheral lesions by a 3 : 1 ratio, and they are usually diagnosed in the second decade of life, while the peripheral ones are usually noted after 50 years [11]. Coinciding with all these reports, our patient was a 20-year-old male, with the lesion presenting in the mandibular third molar region.

CCOT are generally a unilocular lesion, while in 5–13% of cases they are multilocular [12]. Some have a regular outline with well-demarcated margins. Others may be quite irregular and may have poorly defined margins. Early in their development, they will appear completely radiolucent. As they mature, they develop calcifications that produce a well-circumscribed, mixed radiolucent-radiopaque appearance. Three general patterns of radiopacity are seen. One is a salt and pepper pattern of flecks, the second is a fluffy cloud-like pattern throughout, and the third is a crescent-shaped pattern on one side of the radiolucency in a “new moon”-like configuration [4].

CCOT is usually intraosseous (70%) and extraosseous, accounts to 16–22%, and seen in individuals over 50 years of age [12]. About 50% of CCOT have been reported as being associated with an unerupted tooth. Displacement of teeth is often seen. Resorption of the roots of adjacent teeth is a frequent finding and is regarded as an important radiological feature [9]. Local expansion sometimes occurs, and perforation of the cortical plate, when present, may be radiologically demonstrable [1]. The present case report showed multilocular radiolucent lesion associated with an impacted 38 which was displaced into the ramus of the mandible, and root resorption was noted in 36 and 37. In the mandible, several cases have crossed the midline, but this is less usual in the maxilla [4, 9]. Most of the peripheral lesions were located in the maxillary or mandibular gingiva or alveolar mucosa anterior to the first molar [1].

The epithelial lining of CCOT has characteristic odontogenic features. The most remarkable feature is the presence of ghost cells. Budding from the basal layer into the adjacent connective tissue and epithelial proliferations into the lumen are frequently seen. In the fibrous wall, there are usually strands and islands of odontogenic epithelium, either in direct contact with the epithelium or separately in the connective tissue. These vary from a few strands to extensive proliferations.

Takeda et al. stated that the cysts arise *de novo, *and Prætorius et al. concluded from their study that substantial evidence existed that the tumor developed from the wall of the cyst [1]. Hence, CCOT often coexist with other odontogenic tumors, such as odontoma, ameloblastoma, ameloblastic fibroma, ameloblastic fibroodontoma, and adenomatoid odontogenic tumor [10, 13]. Central CCOT has been reported with odontoma in 24–35% of cases and with an impacted tooth in 35% of cases, mostly canine [12].

Freedman et al. proposed that the neoplastic cells originated from well-differentiated ameloblasts, and its neural crest origin confers to this cell a pluripotential capacity to undergo terminal differentiation. Starting from this postulate that ameloblasts are stem cells, terminal differentiation is not necessarily required to originate the CCOT neoplastic cell. Praetorious et al. and Buchner et al. believe that CCOT cystic epithelium originates from the reduced enamel organ, from islands of odontogenic epithelium within the tooth follicle, or from the remnants of the odontogenic epithelium in the bone or gingival tissue [14].

Toida classified CCOT into cyst and a neoplasm. Neoplasm is divided into benign and malignant types, and the terminology calcifying ghost cell odontogenic tumor (CGCOT) is used for benign neoplasm. The cyst or non-neoplastic variant accounts to 80–98% of cases. The solid and neoplastic type accounts to 11.5% of cases [12].

 Pretorious et al. (2006) suggested classification of the odontogenic ghost cell lesions as Group 1 (“simple” cysts), Group 2 (cysts associated with odontogenic hamartomas or benign neoplasms), Group 3 (solid benign odontogenic neoplasms with similar cell morphology to that in the COC, and with dentinoid formation, dentinogenic ghost cell tumor), and Group 4 (malignant odontogenic neoplasms with features similar to those of the dentinogenic ghost cell tumor, ghost cell odontogenic carcinoma) [1]. 

The histologic variation of COC has led to different terminologies such as calcifying ghost cell odontogenic tumor (Fejerskov and Krogh, 1972), dentinogenic ghost cell tumor (Preatorius et al., 1981), epithelial odontogenic ghost cell tumor (Ellis and Shmookler, 1986), and odontogenic ghost cell tumor (Colmenero et al., 1990) [2, 10].

 Gorlin et al., Ebling and Wagner, Gold, Bhasker, Komiya et al., and Regezi et al. all believed that ghost cells represent normal or abnormal keratinization. Levy suggested that they represent squamous metaplasia with subsequent calcification caused by ischemia. Sedano and Pindborg thought that the ghost cells represented different stages of normal and aberrant keratin formation and that they were derived from the metaplastic transformation of odontogenic epithelium. Other investigators suggested or implied that ghost cells may represent the product of abortive enamel matrix in odontogenic epithelium [4, 9].

The classification advocated by Hong et al. has two categories for COC associated with ameloblastoma: the ameloblastomatous cystic variant and the neoplastic variant associated with ameloblastoma. The former is characterized by a unicystic structure in which the lining epithelium shows unifocal or multifocal intraluminal proliferative activity that resembles ameloblastoma, although it also contains isolated or clustered ghost cells and calcifications. The ghost cells and dystrophic calcifications are within the proliferative epithelium, which lacks histopathologic criteria as suggested by Vickers and Gorlin and is confined to the cyst lumen. Ameloblastomatous COC occurs only intraosseously. The latter is called ameloblastoma arising from COC (ameloblastoma ex COC). It is characterized histopathologically as comprising few or no ghost cells with calcifications observed in the transformed ameloblastomatous epithelial portion, while the cyst lining of the epithelium contains a considerable number of ghost cells and calcifications [9, 13, 15].

The present report showed the cystic lining containing ghost cells and few areas showed juxta-epithelial hyalinization suggestive of dysplastic dentin. Proliferative odontogenic islands were seen in the connective tissue which also showed some ghost cells. Satellite cysts containing ghost cells were also noted.

 Takeda et al. (1990) had performed a histological study of satellite cysts and odontogenic epithelial islands in the connective tissue and grouped them into the same three histological types described by Prætorius et al. (1981), but these did not always coincide with the typing of the mother cyst [1].

 Gorlin et al. considered the appearance of dentinoid material in CCOT to represent an inflammatory response of the body tissue in response to ghost cells. Abraham and Howell further stated that masses of ghost cells induced granulation tissue to lay down juxta-epithelial osteoid which may calcify. Contrary to this interception, Sauk stated that the juxta-epithelial osteoid and dentinoid are frequently found in areas free of either granulation tissue or ghost cells and postulated that it might be an inductive phenomenon. To date, it still remains to be clarified that dentinoid is an inductive phenomenon or metaplastic change in connective tissue [14].

Melanin deposits are sometimes present in the epithelial linings. One case, recorded by Gorlin et al. (1964), was found in the parotid salivary gland [1]. CCOT was seen associated with an orthokeratinized odontogenic cyst [6].
